# Copper-Induced Interactions of Caffeic Acid and Sinapic Acid to Generate New Compounds in Artificial Biological Fluid Conditions

**DOI:** 10.3390/antiox11071307

**Published:** 2022-06-30

**Authors:** Yusuke Iwasaki, Rie Manabe, Mika Kimoto, Mao Fukuda, Narumi Mase, Mako Miyazawa, Kotomi Hosokawa, Junzo Kamei

**Affiliations:** 1 Laboratory of Biopharmaceutics and Analytical Science, Hoshi University School of Pharmacy and Pharmaceutical Sciences, 2-4-41 Ebara, Shinagawa-ku, Tokyo 142-8501, Japan; s161246@hoshi.ac.jp (R.M.); s151093@hoshi.ac.jp (M.K.); s151207@hoshi.ac.jp (M.F.); s161241@hoshi.ac.jp (N.M.); s161518@hoshi.ac.jp (M.M.); s131212@hoshi.ac.jp (K.H.); kamei@hoshi.ac.jp (J.K.); 2 Department of Biomolecular Pharmacology, Hoshi University School of Pharmacy and Pharmaceutical Sciences, 2-4-41 Ebara, Shinagawa-ku, Tokyo 142-8501, Japan; 3 Juntendo Advanced Research Institute for Health Science, Juntendo University, 2-1-1 Hongo, Bunkyo-ku, Tokyo 113-8421, Japan

**Keywords:** antioxidant activity, hydroxycinnamic acids, interaction, metal ions, prooxidant activity

## Abstract

Active ingredients may be ingested through foods, and they can cause several interactions in the human body. Although drug–drug or drug–food interactions are evaluated before the approval of medicines, several functional food interactions are not well-documented because of the wide range of possible combinations of interactions. In this study, we examined the chemical reactions between hydroxycinnamic acids (HCAs), a group of polyphenols, and metal ions in artificial gastric juice or artificial intestinal fluid. Caffeic acid (CaA) and sinapic acid (SA) reacted with copper ions under artificial intestinal fluid conditions and produced new compounds. The triple interactions of CaA or SA with iron and copper ions were also examined. Relative to the initial compounds, CaA and SA derivatives produced by condensation exhibited an increased antioxidant and a decreased prooxidant activity. This study revealed a new food ingredient interaction pattern in which new compounds are produced under biological conditions.

## 1. Introduction

Oxidative stress is manifested by excessive reactive oxygen species (ROS) production in the face of insufficient or defective antioxidant defense systems. Oxidative stress which causes profound alterations of various biological structures is considered to be involved in various diseases such as diabetes, Alzheimer’s disease, and cancer [1,2]. It was said that numerous types of functional foods improve human health and can prevent several diseases. Particularly, functional materials such as polyphenols (e.g., catechin, chlorogenic acid, and resveratrol), abundantly found in plants, are widely consumed in the form of dietary supplements and beverages worldwide [3,4,5]. Polyphenols exhibit antioxidant activity, which reduces the excess generation of ROS (by binding metal ions) and prevents damage (such as that caused by radical scavenging) to the human body. Therefore, it is suggested that polyphenols should be taken to prevent diseases involving ROS.

Hydroxycinnamic acids (phenylpropanoic acids: HCAs), a major category of phenolic acids, include caffeic acid (CaA), chlorogenic acid (ChA), sinapic acid (SA), and other related compounds, which are found in plants and plant products, such as coffee, fruits, and vegetables [6,7,8]. Owing to their antioxidant, anti-inflammatory, antidiabetic, and anticancer activities, these compounds are used not only in food and cosmetic materials but also in pharmaceutical and agricultural industries as precursors of active ingredients [9,10]. Particularly, CaA and its derivatives have been applied in combination with antibiotics or photoirradiation to achieve a synergistic mode of action [11]. However, CaA has been classified as a 2B agent (possibly carcinogenic to humans) by the International Agency for Research on Cancer (IARC). Thus, HCAs seem to have both advantages and disadvantages depending on their applications.

On the other hand, metal ions present in several foods and supplements are essential for metabolic reactions and the maintenance and growth of the human body. For example, iron forms complexes and cofactors with several functional proteins, such as hemoglobin and cytochromes; meanwhile, copper, an important metal ion present in chromatin, is closely associated with DNA bases [12]. Moreover, copper supplementation is considered as a potential therapeutic tool for the treatment and prevention of involutional osteoporosis. However, via the Fenton reaction, iron and copper can also react with hydrogen peroxide to produce ROS, i.e., the hydroxyl radical and superoxide radical of highly reactive molecules [13,14].

Although there are many kinds of nutritional ingredients and related compounds to promote human health, dietary supplements or foods may interact with drugs, which could pose some clinical concerns. Therefore, at the early stages of drug development, several in vitro/in vivo/in silico approaches are applied to predict the effects of foods on drugs [15]. Although these interactions are evaluated before the approval of any medicine, several functional food interactions become difficult to evaluate owing to the numerous possible combination patterns. Phenolic compounds act as reducing agents under in vitro conditions and as pro-oxidants in the presence of metal ions, such as copper or iron [16,17]. Furthermore, in vitro, ChA and CaA are nitrated by NaNO_2_ under acidic conditions and produce several nitrated ChA and CaA compounds [18]. On the upside, it has also been reported that whey proteins and polyphenols can be exploited to reduce astringency or increase the solubility and stability of bioactive compounds in foods [19].

Understanding the mechanism of the interactions between dietary elements, supplements, and medicines is essential for ensuring their safety and effectiveness. For this purpose, we examined the chemical reactions between HCAs and metal ions in fasted state simulated gastric fluid (FaSSGF) and fasted state intestinal fluid (FaSSIF). Moreover, we assessed the antioxidant activities of HCAs using 1,1-diphenyl-2-picrylhydrazyl (DPPH), cupric reducing antioxidant capacity (CUPRAC), and ferric reducing antioxidant power (FRAP) assays. We also investigated the prooxidant activities of the newly identified HCAs by electron spin resonance (ESR).

## 2. Materials and Methods

### 2.1. Reagents and Chemicals

Cinnamic acid and its derivatives, such as trans-cinnamic acid (CiA), trans-p-coumaric acid (CoA), coumarin (Cou), ChA, and 3,5-dimethoxy-4-hydroxycinnamic acid (SA) were purchased from Tokyo Chemical Industry (Tokyo, Japan). CaA and trans-4-hydroxy-3-methoxycinnamic acid (ferulic acid; FA) were purchased from Fujifilm Wako Pure Chemical Industries (Tokyo, Japan). Magnesium chloride, calcium chloride, zinc chloride, iron (III) chloride, and copper (I) chloride were purchased from Fujifilm Wako Pure Chemicals Industries. Copper (ΙΙ) sulfate pentahydrate and ammonium iron (II) sulfate hexahydrate were purchased from Kanto Chemical (Tokyo, Japan). 1,1-Diphenyl-2-picrylhydrazyl (DPPH), neocuproine, and 2,4,6-tris(2-pyridyl)-1,3,5-triazine (TPTZ) were purchased from Tokyo Chemical Industry (Tokyo, Japan). FaSSIF/FeSSIF/FaSSGF powders, FaSSGF buffer, and FaSSIF buffer were obtained from Biorelevant.com (London, UK). The biorelevant simulated media, which are USP compendia simulated gastric and intestinal fluids without enzymes were used as reaction media. α-(4-Pyridyl-1-oxide)-N-tert-butylnitrone (POBN) was used as a spin trapping reagent and was purchased from Tokyo Chemical Industry. Water was purified using a Milli-Q Gradient A10 system (Millipore, Billerica, MA, USA). Other chemicals and solvents were obtained from Fujifilm Wako Pure Chemical Industries.

### 2.2. Analytical Conditions of Cinnamic Acids 

Chromatographic analysis using high-performance liquid chromatography/photodiode array (LC/PDA) was performed on a Shimadzu HPLC system (Shimadzu, Kyoto, Japan), consisting of a pump (LC-20AD), autosampler (SIL-20AC_HT_), thermostatic column compartment (CTO-20AC), and photodiode array detector (SPD-M20A). The analytes were separated on a Sunshell C18 column (2.6 μm, 2.1 × 150 mm; ChromaNik Tech., Osaka, Japan). The column oven temperature was maintained at 40 °C. The mobile phase was 0.1% aqueous formic acid (A) and 0.1% formic acid in acetonitrile (B). The gradient elution program was as follows: 0–15 min, 10–30% B; 15–15.1 min, 30–90% B; 15.1–19 min, 90% B; and 19–19.1 min, 90–10% B; followed by the re-equilibration of the initial solvent composition for 10.9 min (19.1–30 min). The flow rate was kept constant at 0.3 mL/min. The photodiode array detector was set in the wavelength range of 200–400 nm.

To identify new cinnamic acid molecules, mass spectra were obtained using Waters Acquity ultra-performance liquid chromatography (UPLC) coupled with a Quattro Premier XE tandem quadrupole mass spectrometer (MS) equipped with an electrospray ionization (ESI) source (Waters Corporation, Milford, MA, USA). The separation conditions were the same as those used for the LC/PDA. The ESI source was operated in the negative ionization mode. The optimal MS conditions were set as follows: capillary voltage, 3.0 kV; source and desolvation temperatures, 120 °C and 400 °C, respectively; desolvation and cone gas flows, 600 L/h and 50 L/h, respectively; and cone voltage, 30 V.

### 2.3. Sample Preparation of Hydroxycinnamic Acids and Metal Ions for the Assessment of Stability

Each HCA (1 mmol/L, 50 μL) and each metal ion (Na^+^, Mg^2+^, K^+^, Ca^2+^, Fe^2+^, Fe^3+^, Cu^+^, Cu^2+^, or Zn^2+^) (1 mmol/L, 50 μL) was reacted under 350 μL of FaSSGF at pH 1.2 or FaSSIF at pH 6.8. Mixed samples were vortexed and incubated at 37 °C for the examination period. Subsequently, HCl (1 mol/L, 50 μL) was added to stop the reaction, and the samples were analyzed by LC/PDA.

### 2.4. Extraction and Purification of CaA and SA Derivatives for the Identification of Their Structure

Derivatives of CaA and SA were extracted and purified using the following method. CuSO_4_·5H_2_O (62 mg, 0.25 mmol) was added to a stirred solution of CaA (45 mg, 0.25 mmol) in methanol (25 mL) and borate buffer (50 mmol/L, pH 9, 200 mL). This reaction mixture was stirred at 40 °C for 30 min, quenched with 1 mol/L HCl, and finally extracted using ethyl acetate. The organic phase was separated, dried over Na_2_SO_4_, and concentrated to dryness. The crude product was purified by liquid chromatography (LC) on an ODS column (eluent: water/acetonitrile/formic acid 70:30:0.1) to obtain the standard reagent.

CuSO_4_·5H_2_O (74.9 mg, 0.30 mmol) was added to a stirred solution of SA (67.2 mg, 0.30 mmol) in methanol (30 mL) and phosphate buffer (50 mmol/L, pH 7.4, 240 mL). The reaction mixture was stirred at 50 °C for 1.5 h, quenched with 1 mol/L HCl, and finally extracted using ethyl acetate. The organic phase was separated, dried over Na_2_SO_4_, and concentrated to dryness. The crude product was purified using LC on an ODS column (eluent: acetonitrile/water/formic acid 40:60:0.1).

The purified compounds were identified by comparing their spectral properties (^1^H NMR, ^13^C NMR, LC/PDA, and LC/MS) with previously published data [20,21,22].

### 2.5. Determination of Antioxidant Activities

Antioxidant activity of HCAs and related compounds was evaluated by DPPH radical scavenging, CUPRAC, and FRAP assays [23,24,25]. All samples of the assay were analyzed by an iMark microplate reader (Bio-Rad, Hercules, CA, USA). Each HCAs were diluted with methanol to 0.1 mmol/L (CaA, ChA, FA, SA, CaA-F1, SA-F1, and SA-F2), 1 mmol/L (Cou), and 10 mmol/L (CiA and CoA), respectively.

The modified DPPH method was used to determine antioxidant activity. A DPPH radical solution (1 mmol/L) was prepared in ethanol. The prepared DPPH (200 μL) solution was added to the diluted sample solution of HCAs (40 μL) and methanol (160 μL). The mixed samples were incubated for 30 min at 37 °C. Absorbance was monitored at 520 nm.

The CUPRAC assay was performed as per the following method. Each HCA solution (50 μL) was added to following reagent containing 20 mmol/L CuSO_4_ (50 μL), 15 mmol/L neocuproine (50 μL), and 0.5 mol/L NH_4_Ac (200 μL). These samples were then incubated for 30 min at room temperature, and their absorbance was monitored at 450 nm.

The FRAP assay was executed according to the following method. The HCAs analyte solution (20 μL) was added to the premixed FRAP reagent (180 μL) containing 0.3 mol/L acetate buffer (pH 3.6), 10 mmol/L TPTZ, and 20 mmol/L FeCl_3_ in a ratio of 10:1:1 (*v*/*v*/*v*). The absorbance of these samples was monitored at 595 nm after their incubation for 10 min at room temperature.

The Trolox equivalent antioxidant capacity (TEAC) was expressed as mmol Trolox equivalent per mmol antioxidant compound, calculated from the produced Trolox standard curve. All measurements were conducted in triplicate.

### 2.6. Electron Spin Resonance (ESR) Condition for Measurement of Hydroxyl Radical

Prooxidant activities of the HCAs and related compounds were determined using our previously reported ESR methods [18,26]. Instead of directly trapping the hydroxyl radical (•OH), this method actually traps and measures the methyl radicals (•CH_3_) using POBN. •CH_3_ are formed from the interactions of DMSO with •OH. The analysis of POBN-CH_3_ was carried out with an ESR spectrometer (JES-RE1X, JEOL Co., Tokyo, Japan). The ESR spectrum was measured at a microwave frequency of 9.43 GHz, a magnetic field of 336.0 ± 5 mT, a microwave power of 9 mW, a modulation frequency of 100 kHz, a time constant of 0.03 s, and a sweep time of 30 s. The spectra of the samples were scanned to record the signal intensities (peak-to-peak heights).

A typical reaction mixture for incubation contained phosphate buffer saline, POBN (10 mM), DMSO (10%), the solution of each HCA (1 mM), and copper (ΙΙ) sulfate pentahydrate (1 mM) in a final volume of 0.3 mL. The reaction of the samples was performed at 37 °C for 30 min.

### 2.7. Statistical Analysis

All results were presented as the mean ± SD of three independent experiments. Most of the data were analyzed using a two-tailed Student’s t-test or one-way ANOVA with Dunnett’s test for pairwise comparisons. The differences were considered significant if the *p*-value was <0.05. All statistical analyses were performed using the Ekuseru-Toukei 2015 software package (Social Survey Research Information Co., Ltd., Tokyo, Japan).

## 3. Results and Discussion

### 3.1. Reaction of HCAs with Metal Ions in Artificial Gastric Juice or Artificial Intestinal Fluid

Because HCAs and related compounds have similar structures and polarities, it was difficult to separate each analyte by conventional LC column which are silica-based column. In the first series of experiments, the LC/PDA method applied core-shell column was examined for the separation of each HCA (1 mmol/L) and the new compounds, which were generated from the interactions of HCAs and metal ions. Although the core-shell column exhibits weak retention under the same LC conditions relative to a silica-based column, it has several merits, such as good resolution and peak-shaped chromatograms [27]. By using a core-shell column instead of a silica-based column, each compound was successfully separated and accurately quantified. The UV spectra and chromatograms of the HCA standards are shown in Figure S1.

Several media have been proposed for simulating the contents of stomach and intestine. Among these, FaSSGF and FaSSIF are one of the most complex media that contain phospholipids and bile salt. The interactions in these stomach or intestinal fluids can significantly influence the reaction of HCAs with nine kinds of metal ions such as Na^+^, K^+^, Mg^2+^, Ca^2+^, Fe^2+^, Fe^3+^, Cu^+^, Cu^2+^ and Zn^2+^. Prior to investigating the interaction of each HCA, we performed the preliminary experiments of five metal ions, including Na^+^, K^+^, Mg^2+^, Ca^2+^, and Zn^2+^ in FaSSGF or FaSSIF. Because the HCAs did not reduce the peak area in LC/PDA chromatograms, HCAs were stable in both FaSSGF and FaSSIF even in the presence of Na^+^, K^+^, Mg^2+^, Ca^2+^, and Zn^2+^. 

**Figure 1:**
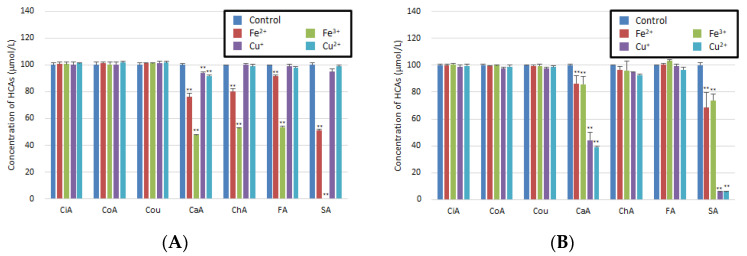
Interaction of HCAs with different metal ions (Fe^2+^, Fe^3+^, Cu^+^, or Cu^2+^). (**A**) HCAs with metal ions in FaSSGF. (**B**) HCAs with metal ions in FaSSIF. Results are presented as the mean ± SD for three independent experiments. ** indicate significantly different values at *p* < 0.05 and *p* < 0.01, respectively.

**Figure 2:**
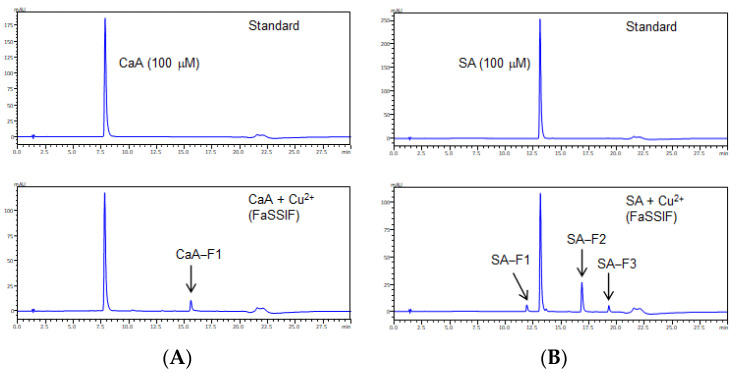
Chromatograms of (**A**) CaA and (**B**) SA with copper ion in FaSSIF after 60 min of reaction. The analysis of CaA and SA for was performed at 320 nm.

In contrast, CaA, ChA, FA, and SA reacted with Fe^2+^ or Fe^3+^, as demonstrated by the significant decrease in the concentration of each compound after incubation in FaSSGF (Figure 1A). CaA and SA decomposed after reacting with Cu^+^ or Cu^2+^ ions in FaSSIF; moreover, they led to the occurrence of new peaks for CaA-F1, SA-F1, SA-F2, and SA-F3 (Figure 1B and Figure 2). However, some limitations should be noted. As there are a lot of different ingredients in foods, we need to examine whether the same interaction occurs under conditions where there are other coexisting substances.

### 3.2. Identification of New Compounds by Nuclear Magnetic Resonance (NMR) Spectroscopy and MS

**Figure 3:**
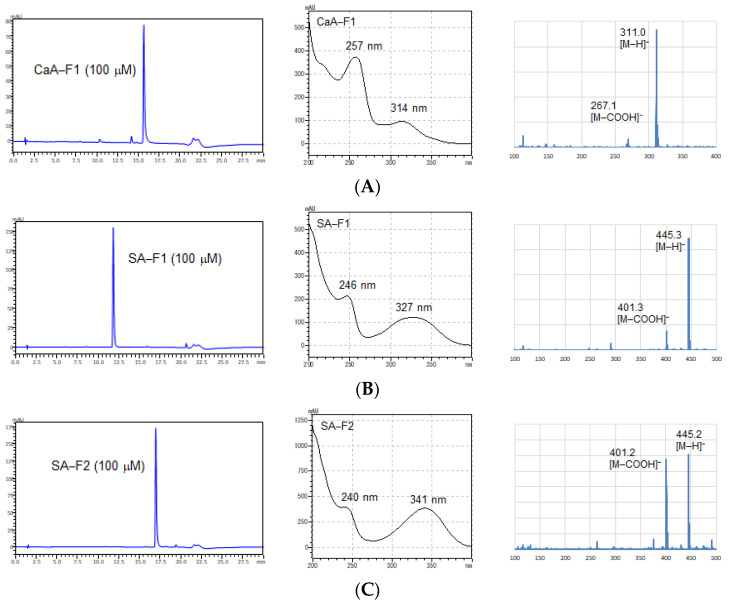
(**Left**) Chromatograms, (**center**) wavelengths, and (**right**) mass spectra of (**A**) CaA-F1, (**B**) SA-F1, and (**C**) SA-F2.

**Figure 4:**
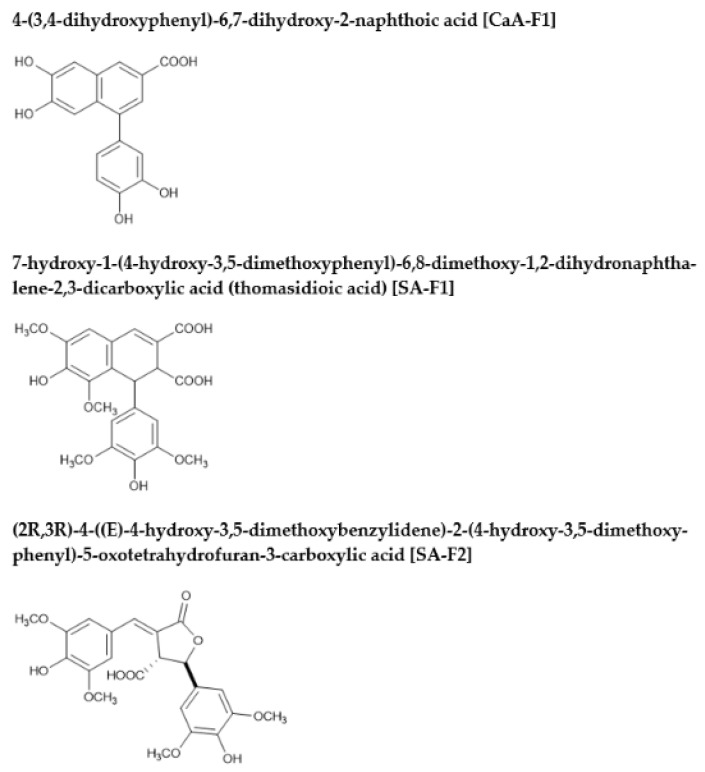
Chemical structures of the synthesized HCAs in reaction mixtures with copper ion.

The structures of the newly formed compounds from the reaction of CaA and SA with Cu^+^ and Cu^2+^ ions in artificial intestinal fluid conditions were identified. The molecular weights of each compound in the reaction mixture were confirmed by MS measurements before being purified by LC/UV. The CaA and SA derivatives exhibited deprotonated ion peaks [M-H]^−^ in their ESI/MS spectra, which appeared at an *m*/*z* of 311.0, 445.3, and 445.2 (Figure 3), suggesting the formation of CaA-F1, SA-F1, and SA-F2, respectively. Moreover, the UV spectra of CaA or SA derivatives using reactions with Cu^+^ and Cu^2+^ ions in FaSSIF did not correspond to those of other HCAs. To identify the interaction products, we extracted CaA-F1, SA-F1, SA-F2, and SA-F3 using LC/UV. After the purification of each product, ^1^H and ^13^C NMR spectroscopy were used to identify the new compounds. CaA-F1 was found to be 4-(3,4-dihydroxyphenyl)-6,7-dihydroxy-2-naphthoic acid, SA-F1 was 7-hydroxy-1-(4-hydroxy-3,5-dimethoxyphenyl)-6,8-dimethoxy-1,2-dihydronaphthalene-2,3-dicarboxylic acid (thomasidioic acid), and SA-F2 was (2R,3R)-4-((E)-4-hydroxy-3,5-dimethoxybenzylidene)-2-(4-hydroxy-3,5-dimethoxyphenyl)-5-oxotetrahydrofuran-3-carboxylic acid (Figure 4). CaA-F1, SA-F1, and SA-F2 were known compounds that are involved in the condensation of the owing two molecules [20,21,22]. Unfortunately, we could not purify and determine the structure of SA-F3 because of its unstable nature.

Literature has reported several methods for the synthesis of CaA-F1 and SA-F1. Several studies have reported that the conversion of monomers (CaA and SA) into dimers requires several reagents and steps for synthesis. However, our method demonstrated the synthesis of dimers such as thomasidioic acid using copper ions in neutral conditions at a pH of 6.8.

### 3.3. Comparison of Reaction Conditions for CaA or SA with Copper Ion

**Figure 5:**
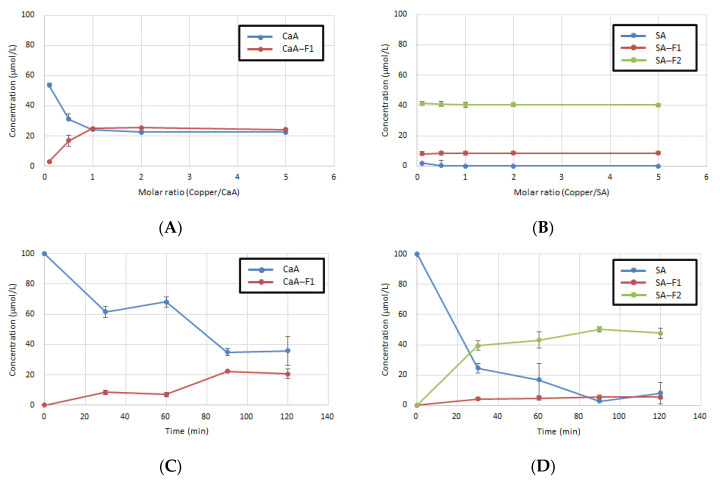
Comparative reaction conditions of CaA or SA with copper ions in FaSSIF. (**A**) Molar ratio of CaA for 60 min at 37 °C; (**B**) molar ratio of SA for 60 min at 37 °C; (**C**) time course of CaA at 37 °C; and (**D**) time course of SA at 37 °C. Results are presented as the mean ± SD for three independent experiments.

The result of the market basket survey in Egyptian fruits and vegetables indicated that their average copper ion concentrations ranged from 0.83 to 18.3 mg/kg [28]. Moreover, in Japan, the daily intake of metals from meals using the market basket method was investigated, which indicated a daily intake of 1.2 mg of copper ions [29]. HCAs, especially CaA, are found in food and beverages, such as coffee, cocoa, apple, and honey. They contain higher concentrations of polyphenols than metal ions. Generally, at the physiological pH (7.2), a mixture of ligand and metal species exists in solutions at the ratios of 2:1 and 3:1, depending on the polyphenol and its stability constant with iron ion (Fe^2+^ and Fe^3+^) [30]. The antioxidant activity of polyphenols increases with the increase in their concentrations. Therefore, we considered the effect of the ratio of HCAs and copper ions on the synthesis of CaA-F1, SA-F1, and SA-F2compounds derived from CaA and SA (Figure 5A,B). All compounds excepted SA-F2 were quantified by LC/PDA with a calibration curve. Although the highest amount of CaA-F1 was produced at a molar ratio of 1:1, SA-F1 and SA-F2 were produced the same concentration at every condition under the examined molar ratio.

Additionally, we examined the reaction time and stability of CaA or SA with copper ions because some food ingredients can remain in the body for a long time. The emptying of food from the stomach is a complex process, as it depends on the breakdown of food, physical properties of the digesta, and physiological regulation. Moreover, the average digestion time is over 3 h in the stomach or intestine [31,32]. In this study, the reaction of CaA and SA with copper ions in FaSSIF was examined for 120 min. The results indicated that the concentration of CaA-F1 and SA-F1 not only increased over time but they also exhibited stability under neutral conditions (pH ≈ 6.8) (Figure 5C,D).

### 3.4. Co-Reaction of CaA or SA with Metal Ions 

**Figure 6:**
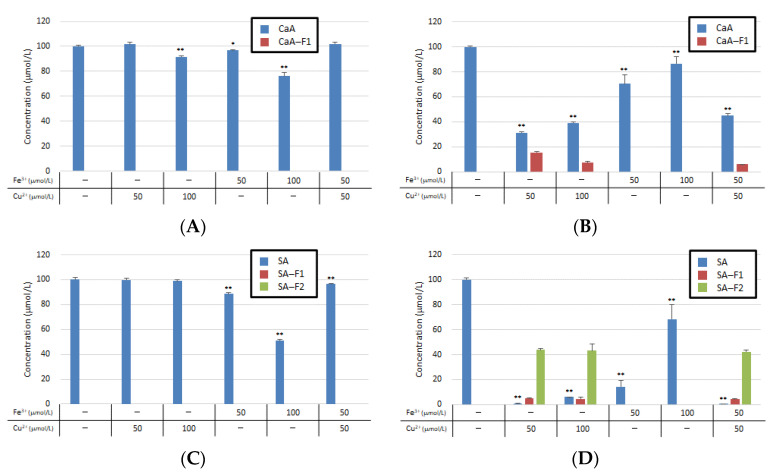
Co-interaction of metal ions with (**A**) CaA in FaSSGF; (**B**) CaA in FaSSIF; (**C**) SA in FaSSGF; (**D**) SA in FaSSIF. * and ** indicate significantly different values at *p* < 0.05 and *p* < 0.01, respectively.

Food interactions can occur in many combinations. Because CaA and SA can react with iron copper ions and can be converted into new compounds in FaSSIF, we examined the triple interaction of CaA or SA individually with Fe^3+^ and Cu^2+^ ions. The Fe^3+^ and Cu^2+^ ions showed additive action but did not exhibit a synergistic effect (Figure 6).

Moreover, a metal chelator and an antioxidant were used to estimate the mechanism of the synthesis reaction. EDTA, a metal chelator, and ascorbic acid, a soluble antioxidant, inhibited the synthesis of the CaA and SA derivatives (Figure S2). Although these enzymes such as catalase and superoxide dismutase inhibited the reaction between CaA and Cu^2+^, they could not inhibit the reaction of SA to generate SA-F1 and SA-F2. The common reaction of these compounds may have occurred due to the presence of Cu^2+^ ion and ROS. Our result supports those of the previous studies because EDTA, a metal chelator, and ascorbic acid could inhibit this reaction. However, enzyme experiments using catalase and superoxide dismutase gave different results for CaA and SA.

### 3.5. Antioxidant and Prooxindat Activity of HCAs and Related Compounds

The antioxidant activities of the HCAs and related compounds were measured by three kinds of antioxidant methods (DPPH, CUPRAC, and FRAP). The scavenging effects of free radicals and the reducing power of metal ions by HCAs are listed in Table 1. Most of the HCAs, except CiA and Cou, exhibited antioxidant activities. The DPPH radical scavenging activities of CaA-F1, SA-F1, and SA-F2 were higher than those of the corresponding HCAs; moreover, they also increased the reducing activities of metal ions. As polyphenols have several functions, it is vital to study the relationship between their structures and functions. Nea et al. reported that the antioxidant potential of polyphenols is controlled by the hydroxyl group (especially the ortho and para bases) in their chemical structure, which is directly linked to an aromatic hydrocarbon ring [33]. Thus, the antioxidant, free radical scavenging, and metal reducing activities of CaA, ChA, and SA were superior to those of the other HCAs. Furthermore, CaA-F1, SA-F1, and SA-F2, which were products of two components, exhibited stronger antioxidant activities because they contained a higher number of OH positions compared to their precursors. 

**Figure 7:**
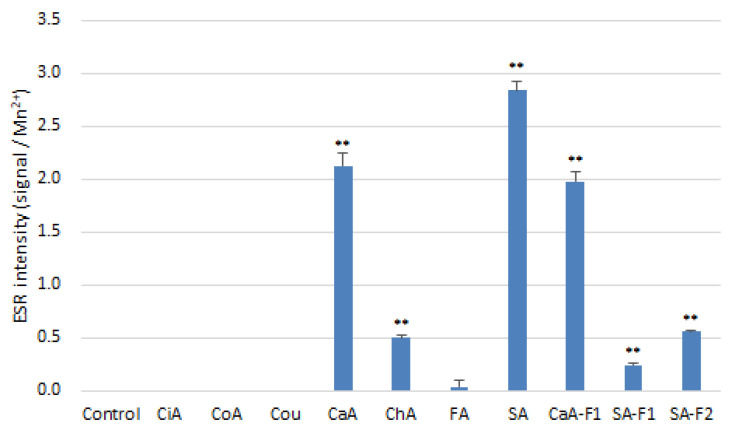
Prooxidant activities produced by the reaction between HCAs and copper ions. Results are presented as the mean ± SD for three independent experiments. ** indicate significantly different values at *p* < 0.05 and *p* < 0.01, respectively.

In our previous study, we demonstrated that compounds bearing o-dihydroxyl groups on benzene rings, such as CaA and ChA, produce ROS in the presence of Cu^2+^ and induce calf thymus DNA oxidation [26]. Therefore, we measured by ESR whether ROS was produced by the reaction of HCA and related compounds with copper (Figure 7). The results indicated that CaA, ChA, and SA, which have ortho dihydroxyl groups in their structures, generated ROS in the presence of Cu^2+^. Although the prooxidant activities of SA-F1 and SA-F2 induced by copper ion decreased, CaA-F1 exhibited the same activity as CaA under the same conditions.

The SA-F1 (thomasidioic acid) present in rice bran reportedly shows a higher antioxidant activity than SA [34]. CaA-F1 and SA-F2 are the dimers of precursor compound; they seem to have a distinct structure, but their properties are not well known yet. As we only examined antioxidant and prooxidant activities in this study, the new products should be further investigated in terms of their positive and negative (such as carcinogenesis) biological effects on human health. However, our results demonstrated that new compounds generated from the reaction of precursor compounds with copper ions have high antioxidant and low prooxidant activities.

## 4. Conclusions

In this study, the combined reactions of HCAs and metal ions were examined under biological conditions. Most HCAs except CiA, CoA, and Cou decreased in concentration upon reacting with iron or copper ions in FaSSGF or FaSSIF. CaA and SA were converted into new compounds upon reacting with copper ions. CaA-F1 and SA-F1, which were the dimer product of CaA and SA, respectively, could obtain a higher yield under the same concentrations of polyphenols and copper ions in FaSSIF. They also exhibited stronger antioxidant activities and lower prooxidant activities than those of their precursors. This study introduced a new possible food ingredient interaction pattern in which new compounds are produced under biological conditions. Our approach may contribute to a better understanding of the interactions between polyphenols and metal ions.

## Figures and Tables

**Table 1:** Trolox equivalent antioxidant capacities (mmol Trolox equivalent/mmol) for various HCAs and related compounds. Results are presented as the mean ± SD for three independent experiments.

HCAs	DPPH	CUPRAC	FRAP
CiA	N.D.	N.D.	N.D.
CoA	0.22 ± 0.003	0.53 ± 0.01	0.19 ± 0.002
Cou	N.D.	N.D.	N.D.
CaA	2.96 ± 0.05	3.31 ± 0.10	1.90 ± 0.04
ChA	3.83 ± 0.62	3.52 ± 0.03	1.59 ± 0.08
FA	1.87 ± 0.44	1.25 ± 0.02	0.99 ± 0.01
SA	1.87 ± 0.13	1.60 ± 0.03	1.71 ± 0.02
CaA-F1	5.00 ± 0.58	4.90 ± 0.09	2.70 ± 0.05
SA-F1	2.42 ± 0.14	3.17 ± 0.04	3.01 ± 0.05
SA-F2	2.42 ± 0.50	2.74 ± 0.05	2.64 ± 0.02

N.D.: Not detected.

## Data Availability

Data are contained within the article and Supplementary Material.

## Supplementary Materials

The following supporting information can be downloaded at: https://www.mdpi.com/article/10.3390/antiox11071307/s1, Figure S1: Chromatograms of standard HCAs, their chemical structures, and their UV spectra showing particular wavelengths; Figure S2: Inhibitation of HCA derivatization using antioxidants or chelators.
