# Supplementary material for: Copper-Induced Interactions of Caffeic Acid and Sinapic Acid to Generate New Compounds in Artificial Biological Fluid Conditions
Source: Antioxidants (Basel). 2022 Jun 30;11(7):1307. doi: 10.3390/antiox11071307 (PMC9311897; doi:10.3390/antiox11071307)

Figure S1: Chromatograms of standard HCAs, (A) their chemical structures, and (B) their UV spectra showing particular wavelengths. Described numbers in spectra are λ_{max} of the wavelengths. The analytes were separated on a Sunshell C18 column (2.6 µm, 2.1 × 150 mm; ChromaNik Tech., Japan). The column oven temperature was maintained at 40 °C. The mobile phase was 0.1 % aqueous formic acid (A) and 0.1 % formic acid in acetonitrile (B). The gradient elution program was as follows: 0–15 min, 10–30 % B; 15–15.1 min, 30–90 % B; 15.1–19 min, 90 % B; and 19–19.1 min, 90–10 % B; followed by the re-equilibration of the initial solvent composition for 10.9 min (19.1–30 min). The flow rate was kept constant at 0.3 mL/min.

(A)

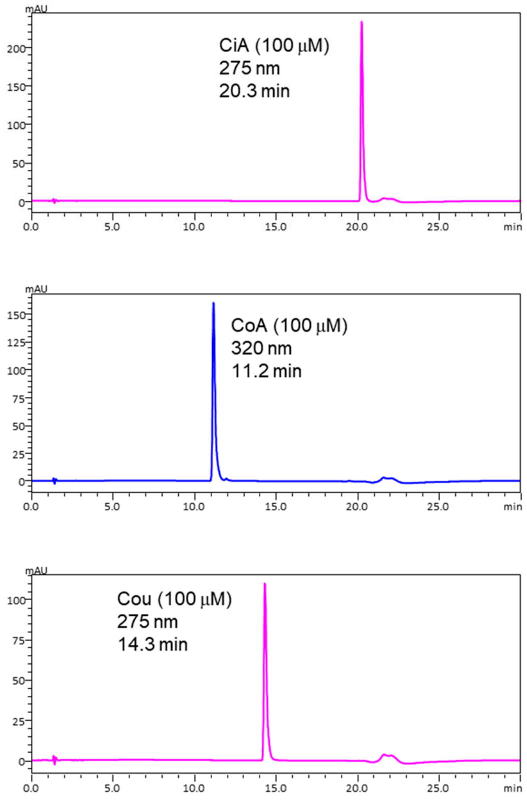

(B)

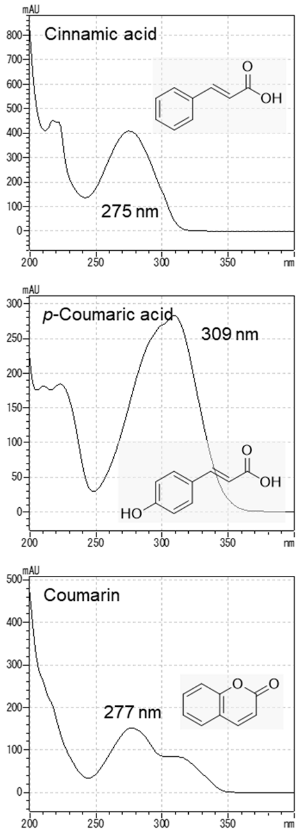

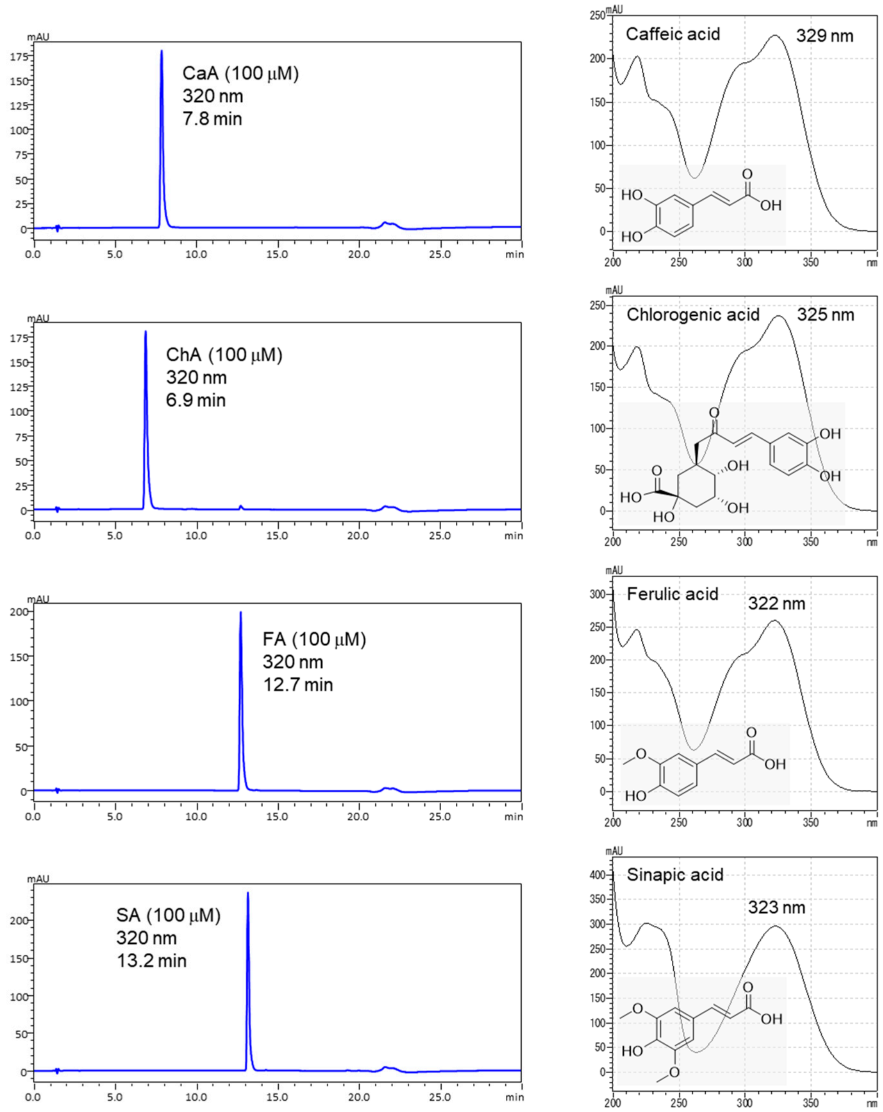
CaA (100 µM)
320 nm
7.8 min
ChA (100 µM)
320 nm
6.9 min
FA (100 µM)
320 nm
12.7 min
SA (100 µM)
320 nm
13.2 min
mAU
min
Caffeic acid
329 nm
Chlorogenic acid
325 nm
Ferulic acid
322 nm
Sinapic acid
323 nm
nm
HO
OH
O

Figure S2: Inhibitation of HCA derivatization using antioxidants or chelators.

(A) CaA with copper ion in FaSSIF at 37 °C

(B) SA with copper ion in FaSSIF at 37 °C

(A)

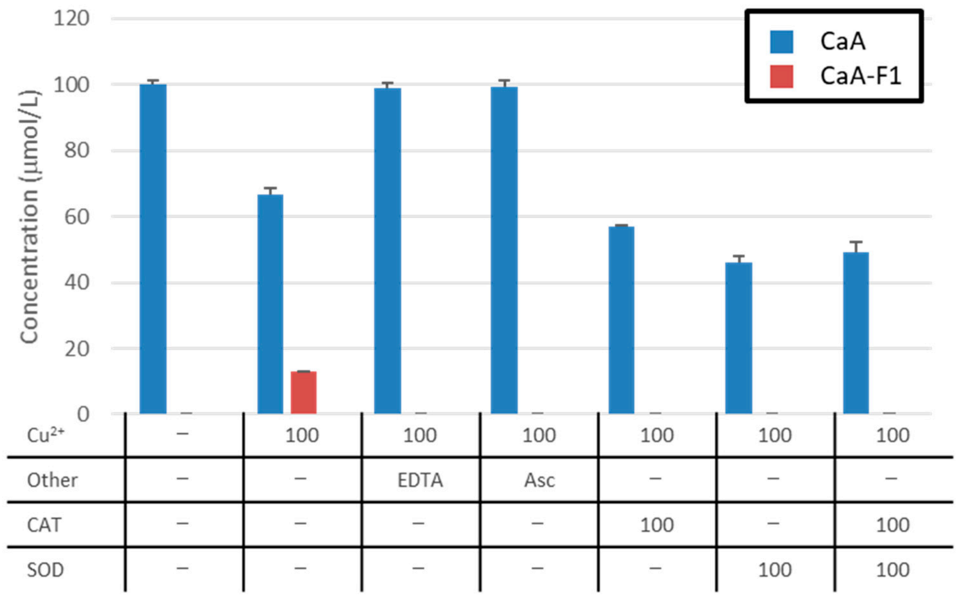

(B)

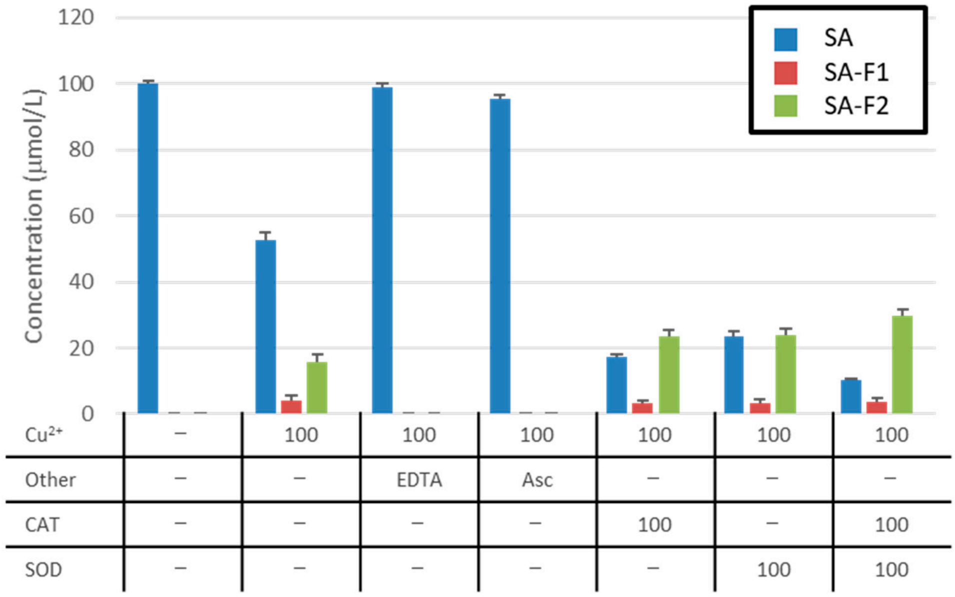

Supplement: Supplementary file 1 [file antioxidants-11-01307-s001.zip › antioxidants-1764019-supplementary.pdf]
